# Rehabilitation Care at the Time of Coronavirus Disease-19 (COVID-19) Pandemic: A Scoping Review of Health System Recommendations

**DOI:** 10.3389/fnagi.2021.781271

**Published:** 2022-01-04

**Authors:** Ahmed M. Negm, Adrian Salopek, Mashal Zaide, Victoria J. Meng, Carlos Prada, Yaping Chang, Preeti Zanwar, Flavia H. Santos, Elena Philippou, Emily R. Rosario, Julie Faieta, Jason R. Falvey, Amit Kumar, Timothy A. Reistetter, Vanina Dal Bello-Haas, Jonathan F. Bean, Mohit Bhandari, Patricia C. Heyn

**Affiliations:** ^1^Faculty of Rehabilitation Medicine, University of Alberta, Edmonton, AB, Canada; ^2^School of Rehabilitation Science, McMaster University, Hamilton, ON, Canada; ^3^Faculty of Health Sciences, McMaster University, Hamilton, ON, Canada; ^4^Faculty of Sciences, McMaster University, Hamilton, ON, Canada; ^5^Division of Orthopaedic Surgery, Department of Surgery, McMaster University, Hamilton, ON, Canada; ^6^OrthoEvidence Inc., Burlington, ON, Canada; ^7^Jefferson College of Population Health, Thomas Jefferson University, Philadelphia, PA, United States; ^8^NIA Funded U.S. Network on Life Course and Health Dynamics and Disparities in the 21st Century America, University of Southern California, Los Angeles, CA, United States; ^9^University College Dublin (UCD), Centre for Disability Studies, School of Psychology, University College Dublin, Dublin, Ireland; ^10^Department of Life and Health Sciences, School of Sciences and Engineering, University of Nicosia, Nicosia, Cyprus; ^11^Department of Nutritional Sciences, King’s College London, London, United Kingdom; ^12^Casa Colina Hospital and Centers for Healthcare, Pomona, CA, United States; ^13^Department of Rehabilitation Science and Technology, University of Pittsburgh, Pittsburgh, PA, United States; ^14^Department of Physical Therapy and Rehabilitation Science, University of Maryland School of Medicine, Baltimore, MD, United States; ^15^Department of Epidemiology and Public Health, University of Maryland School of Medicine, Baltimore, MD, United States; ^16^Center for Health Equity Research, Northern Arizona University, Flagstaff, AZ, United States; ^17^Department of Occupational Therapy, School of Health Professions, University of Texas Health Science Center at San Antonio, San Antonio, TX, United States; ^18^New England Geriatric, Research, Department of PM&R, Harvard Medical School, Education and Clinical Center, VA Boston Healthcare System, Boston, MA, United States; ^19^Spaulding Rehabilitation Hospital, Boston, MA, United States; ^20^Department of Surgery, Faculty of Health Sciences, McMaster University, Hamilton, ON, Canada; ^21^Marymount Center for Optimal Aging, School of Health Sciences, Marymount University, Arlington, VA, United States

**Keywords:** COVID-19, pandemic, rehabilitation, health system, GRADE, SARS-CoV-2, scoping review

## Abstract

**Purpose:** The coronavirus disease-19 (COVID-19) was declared a pandemic by the World Health Organization in March 2020. COVID-19, caused by SARS-CoV-2 has imposed a significant burden on health care systems, economies, and social systems in many countries around the world. The provision of rehabilitation services for persons with active COVID-19 infection poses challenges to maintaining a safe environment for patients and treating providers.

**Materials and Methods:** Established frameworks were used to guide the scoping review methodology. Medline, Embase, Pubmed, CINAHL databases from inception to August 1, 2020, and prominent rehabilitation organizations’ websites were searched.

**Study Selection:** We included articles and reports if they were focused on rehabilitation related recommendations for COVID-19 patients, treating providers, or the general population.

**Data Extraction:** Pairs of team members used a pre-tested data abstraction form to extract data from included full-text articles. The strength and the quality of the extracted recommendations were evaluated by two reviewers using the Grading of Recommendations, Assessment, Development and Evaluation (GRADE) approach.

**Results:** We retrieved 6,468 citations, of which 2,086 were eligible for review, after duplicates were removed. We excluded 1,980 citations based on title and abstract screening. Of the screened full-text articles, we included all 106 studies. A summary of recommendations is presented. We assessed the overall evidence to be strong and of fair quality.

**Conclusion:** The rehabilitation setting, and processes, logistics, and patient and healthcare provider precaution recommendations identified aim to reduce the spread of SARS-CoV-2 infection and ensure adequate and safe rehabilitation services, whether face-to-face or through teleservices. The COVID-19 pandemic is rapidly changing. Further updates will be needed over time in order to incorporate emerging best evidence into rehabilitation guidelines.

## Introduction

The coronavirus disease-19 (COVID-19) was declared a pandemic by the World Health Organization (WHO) in March 2020 (WHO, 2021). As of May 27, 2021, 169,615,273 cumulative cases and 3,524,490 deaths (Ritchie et al., 2020) were reported globally. The United States, with a population of 331.4 million people, continued to have the highest burden of COVID-19 on this date, with 33,999,680 cases and 607,726 deaths (Centers for Disease Control and Prevention, 2020). India, the second-most populous country in the world with 1.4 billion people, had 27,547,705 cases and 318,821 deaths (Ministry of Health and Family Welfare, 2020).

The COVID-19 pandemic caused by SARS-CoV-2 has created a significant burden on healthcare systems, economies, and social systems around the world (WHO, 2021). An ongoing concern with the COVID-19 pandemic is the unknown rate of transmission amongst asymptomatic carriers (Zhao H. et al., 2020). Infected individuals are contagious up to 48 h prior to the development of symptoms (Huff and Singh, 2020). As a result, many countries implemented significant public health requirements that changed the daily practices of their citizens.

Local policies on social distancing, closure of non-essential services, and stay-at-home orders have impacted outpatient medical and rehabilitation access to care. In a survey of individuals with chronic neurologic disorders at a center in Italy, nearly one-third of individuals experienced a delay in medical or rehabilitative care, with 19% of individuals reporting a subjective worsening of symptoms (Piano et al., 2020). Loss of rehabilitation services can lead to a decline in physical function and increased symptoms (Manto et al., 2020; Naser Moghadasi et al., 2020).

The provision of rehabilitation services for persons with active COVID-19 infection poses many challenges to maintaining a safe environment for both patients and treating providers. For example, the required use of immunosuppressant agents in individuals with conditions such as multiple sclerosis and some types of cerebellar ataxias increases susceptibility to severe complications from COVID-19 infections. In certain cases, it may be more judicious to delay rehabilitation admission until patients are no longer at risk of spreading COVID-19 infection to uninfected individuals. The recommended time period for an individual to be considered “no longer at risk” is at least 10 days following symptoms onset and 2–3 days symptom-free after discontinuation of antipyretic medications (Faux et al., 2020; Miles et al., 2021). Based on local health department and hospital protocols, recommendations may also include two separate negative COVID-19 test results on subsequent days (Faux et al., 2020; Miles et al., 2021).

While awaiting resolution of SARS CoV-2 in infected patients, infectivity prevents the timely transfer of patients from the acute care or hospital setting, which then delays rehabilitation care and results in bed shortages in the acute care setting. However, specialized rehabilitation units can meet the needs of those who are currently SARS CoV-2 positive. Prior to admission to these specialized units, patients should be screened to assess whether their physical and medical needs can be met at the rehabilitation facility. Dedicated staff should be allocated to the facility and enhanced personal protective equipment (PPE) such as N95 respirators, face shields for eye protection, gloves and a full-body suit or gown to prevent particle deposition on clothing should be utilized (Levi et al., 2020).

A significant challenge for inpatient rehabilitation facilities has been SARS CoV-2 infection prevention in units that provide care for non-infected individuals. It has been reported that in skilled nursing facilities the risk for infection outbreaks is high due to the large number and variety of individuals who need to be in close contact with patients in order to provide adequate care (Appeadu et al., 2020). Furthermore, persons admitted to rehabilitation facilities often have physical impairments that require caregivers to be educated on how to provide physical assistance at home (Gimm et al., 2017; Appeadu et al., 2020).

The COVID-19 pandemic has also accelerated the need for telehealth strategies such as video calls and applications to facilitate access to medical and rehabilitation care. While these strategies have multiple advantages, several limitations exist. First, patients utilizing telehealth need a prior understanding of how to utilize the technology, making access difficult for cognitively impaired individuals and older adults who may not be technologically savvy (Levi et al., 2020; Sahu and Rathod, 2020). Second, data safety and privacy are of concern with the use of telehealth services (Cottrell and Russell, 2020; Scherrenberg et al., 2020), and can impose barriers for utilization by both the patient and the practitioner when adequate training has not been provided (Deverell et al., 2020). These necessitate the use of unfamiliar applications to facilitate the secure exchange of medical information. Third, costs, including the initial infrastructure to support telehealth services and decreased or absent reimbursement (Besnier et al., 2020; Cottrell and Russell, 2020), are a frequent barrier to delivering telehealth services. Additionally, affordability of devices to access telehealth can be a concern for patients residing in poorer regions (Deverell et al., 2020). Fourth, internet accessibility and connectivity can limit usability (Caze Ii et al., 2020; Polgar et al., 2020; Sahu and Rathod, 2020; Salawu et al., 2020), particularly in less populated areas. Fifth, there is a lack of scientific evidence demonstrating the efficacy of telehealth strategies for rehabilitation treatment (Salawu et al., 2020; Miles et al., 2021). While patients have reported high levels of satisfaction when utilizing telehealth for musculoskeletal physiotherapy (Cottrell and Russell, 2020), there is a need for further research into the use of telehealth strategies. The development of a successful telehealth program requires extensive work at both the early development stage (determining materials, assessments, communication functions) and the transition to a format suitable for telehealth (Caze Ii et al., 2020; Cottrell and Russell, 2020). Finally, few telehealth guidelines for rehabilitation professionals exist.

Due to the rapidly changing impact of the COVID-19 pandemic on healthcare systems globally, and the significant necessity for timely and accessible rehabilitation services, there is a critical need for wide-scale and generalizable rehabilitation-related recommendations. In response to the global pandemic, we launched a COVID-19 task force in the American Congress of Rehabilitation Medicine (ACRM) to help address the lack of contemporary research assessing the impact of COVID-19 on rehabilitation. The task force is a cross-national and multidisciplinary team of clinicians and researchers with diverse rehabilitation and health services expertise across care settings. This report presents system-related rehabilitation recommendations to address the current COVID-19 pandemic and future outbreaks that may affect the delivery of rehabilitation services. More specifically, these recommendations were formulated to support rehabilitation care for individuals with complex healthcare needs and functional limitations who are at higher risk for contracting COVID-19, as well as COVID-19 survivors. We include infection prevention and PPE recommendations, while acknowledging facility-specific and local health policies for mitigating COVID-19 spread (Gimm et al., 2017; Levi et al., 2020; Miles et al., 2020; Salawu et al., 2020).

## Materials and Methods

We utilized the framework proposed by Arksey and O’Malley (2005) and Levac et al. (2010) to guide our scoping review methodology. The Preferred Reporting Items for Systematic Reviews and Meta-Analyses (PRISMA) Extension for Scoping Reviews (PRISMA-ScR) guidelines were followed to ensure consistency and high quality of research reporting (Tricco et al., 2018).

### Development of Research Questions

Our main concept of interest was rehabilitation care (including physiotherapy, occupational therapy, speech-language pathology, physiatry, psychology and other rehabilitation professions) during the COVID-19 pandemic. Our outcomes of interest were rehabilitation related recommendations from health systems, without restriction to country, based on expert opinion, consensus, or research data.

### Identifying Relevant Studies

A health science librarian conducted a comprehensive literature search using the Medline, Embase, Pubmed, CINAHL databases (from inception to August 1, 2020), and identified though rehabilitation organizations’ websites. Study team members conceptualized the search strategy based on the COVID-19 pandemic and rehabilitation concepts, with multiple text words and subject headings (e.g., MeSH) describing each concept. The search strategy was limited to full-text articles in English (see Supplementary Material).

### Selection Criteria

Manuscripts/reports were included if they focused on rehabilitation recommendations for addressing COVID-19, COVID-19 survivors, or the general population at the time of the COVID-19 pandemic.

### Screening and Study Selection

Search results were uploaded to the Covidence platform (Covidence, 2014). After removing duplicates, four team members (MZ, AS, VM, AN) were paired and independently reviewed the titles and abstracts using the inclusion criteria. If there were insufficient details to make an informed decision on an article, the article was retrieved for review. To confirm eligibility, two team members (MZ, AS, VM, AN) independently assessed the full-text articles using the same inclusion criteria. Any disagreement was resolved through consensus or third-party adjudication (AN).

### Data Extraction

A standardized data abstraction form was created and pre-tested. Team members in pairs (MZ, AS, VM, AN) then used the pre-tested data abstraction form to abstract data from included full-text articles.

### Quality Assessment

**FIGURE 1:**
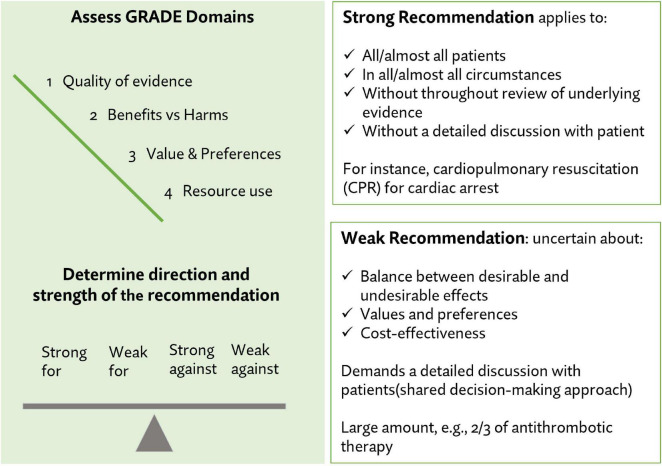
Strength and quality assessments for individual recommendations.

The strength and the quality of the extracted recommendations were evaluated by two reviewers (MZ, VM) using the Grading of Recommendations, Assessment, Development and Evaluation (GRADE) approach (Andrews et al., 2013; Neumann et al., 2014). The strength of the recommendation evidence included four possible categories: (1) Strong recommendation for, (2) weak recommendation for, (3) weak recommendation against, or (4) strong recommendation against. Figure 1 shows the GRADE strength categories and outlines the clinical application of recommendations based on level of strength. Three categories were used to assess the quality of recommendation: (1) Good, (2) fair, and (3) poor. The quality and strength of the extracted recommendations are presented in the results.

### Summarizing and Reporting the Findings

We organized the extracted recommendations into several sections. For each section, a summary of contributing studies, along with the strength and the quality of recommendations are reported.

## Results

**FIGURE 2:**
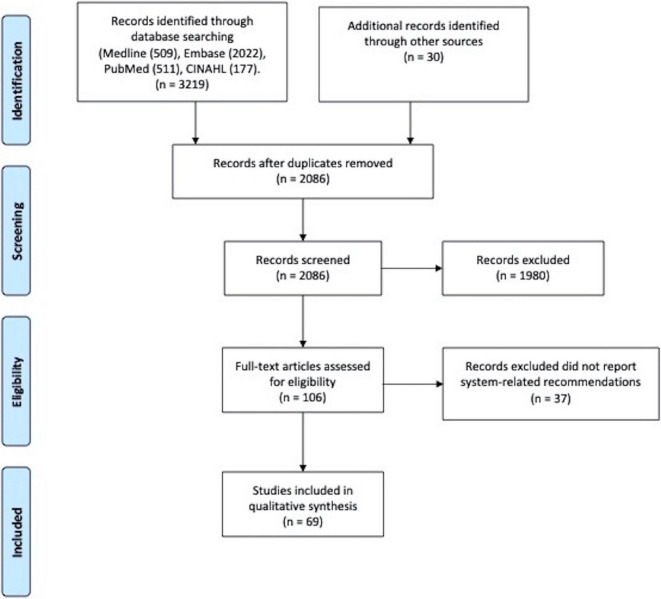
PRISMA flow diagram.

We retrieved 6,468 citations, of which 2,086 were eligible after duplicates were removed. Of those, 1,980 citations were excluded based on the title and the abstract screening. Of the screened full-text articles, 106 studies from 22 countries (including low-income, middle-income and high-income) reported COVID-19 related recommendation (Figure 2). Of these articles, 69 articles reported health system-related recommendations.

### The Extracted Recommendations

**FIGURE 3:**
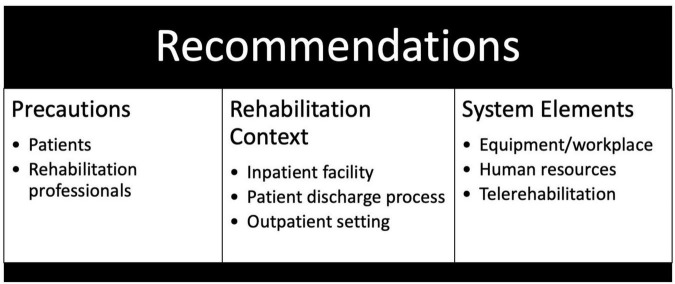
Structure of health system recommendations.

A set of health system focused recommendations related to the COVID-19 pandemic is presented. The recommendations were grouped as follows: (1) recommendations for the rehabilitation inpatient facility setting, the discharge process and the outpatient setting; (2) recommendations related to health system elements e.g., rehabilitation equipment/workplace, human resources and telerehabilitation; and (3) precautions for patients and rehabilitation professionals (see Figure 3).

### Quality and Strength of the Recommendations

Based on the GRADE approach for evidence quality assessment, we have determined the overall evidence for the recommendations included in our report to be strong and of fair quality (Table 1). The strength of each recommendation is reported in Supplementary Table 1.

**TABLE 1:** Recommendations quality.

Standard	Rating
Establishing transparency	Fair
Management of COI* in the guideline development group	Fair
Recommendation development group composition	Good
Recommendation development (evidence-based)	Fair
Establishing evidence foundations and rating strength for each of the recommendations	Fair
Articulation of recommendations	Fair
External review	Not reported
Updating	Fair
Implementation issues	Not reported

*COI, Conflict of interest.

### Rehabilitation Inpatient Facility

Ten articles that addressed recommendations for rehabilitation inpatient facility, published from April 13, 2020, to August 6, 2020 (Bartolo et al., 2020; Boldrini et al., 2020; Grabowski and Joynt Maddox, 2020; Juárez-Belaúnde et al., 2020; Mammi et al., 2020; Pedersini et al., 2020; Qu et al., 2020; Sheehy, 2020; Simpson and Robinson, 2020; Sonel Tur and Evcik, 2020; Tur and Evcik, 2020), were identified. Six countries—Spain (*n* = 1), Italy (*n* = 4), China (*n* = 1), Turkey (*n* = 1), Canada (*n* = 2), US (*n* = 1), and 10 different institutions, including hospitals, scientific societies, and universities were represented (see Supplementary Table 1).

The recommendations found in these 10 articles were based on expert opinion and/or clinical experience. Expertise included the following: researchers (*n* = 3), rehabilitation and medical professionals (*n* = 5), and unknown (*n* = 2, did not report the group involved in developing the recommendations).

### Summary of Key Recommendations

• Control the entrance of patients to rehabilitation institutions.
• Standardize pre-examination triage points at outpatient and emergency departments.
• Formulate institutional screening procedures for patient admission during the pandemic in accordance with the risk level of the country and region.
• Expedite screening and diagnosis of patients with suspected infection, implement isolation measures quickly, and shorten the time between diagnosis and hospitalization.
• Post notices of behavioral rules at the entrances of, and within all departments.
• Post recommendations for hand hygiene near hand-sanitizing gel dispensers.
• Limit admissions to rehabilitation wards to essential admissions, provide oversight to ensure essential admissions only, and prepare personnel with protective equipment. Close access to all other rehabilitation wards.
• Treat patients in individual rooms before SARS-CoV-2 infection is ruled out. Transfer patients to a conventional ward for further hospitalization after ruling out SARS-CoV-2 infection.
• Proactively monitor nosocomial infections in hospitalized patients to establish health monitoring and a mandatory reporting system for all hospital personnel, including all medical, nursing, management, logistics, cleaning, security, delivery, and other staff.
• Caregiver visits to hospitalized patients to be authorized by clinicians according to the rules of health management. Staff to manage access to avoid any physical contact, even for a limited time.
• Screen and manage escorts and visitors as necessary. Monitor body temperature for all health personnel and visitors with permission. Body temperature should be ≤ 37.5°C.
• Prohibit access and provide indication for home isolation for individuals with higher temperature.
• Offer virtual visits.
• Suspend all in-person meeting activities and replace them with telephone, email, or other virtual meeting tools.
• Suspend all rehabilitation activities that require internal flow (movement between floors or between floors and gym) for patients with COVID-19.
• Transition all non-essential treatments to a telerehabilitation/virtual reality modality. Manage clinical cases through telephone or webcam counseling to supervise exercise sessions that can be temporarily self-managed by the patient or caregiver.
• When viral variants of COVID-19 are present, evaluate the reintroduction of certain contact situations with appropriate PPE and devices for circumstances that require urgent hands-on treatment to protect the patient from harmful consequences (e.g., hypo-mobility, respiratory dysfunction, or contextual factors).

### Patient Discharge Process

Recommendations related to patient discharge were based on six articles, all of which were clinical experience or opinion articles (Alberta Health Services, and Scientific Advisory Group Representative, 2020; Grabowski and Joynt Maddox, 2020; Kemps et al., 2020; Pinto and Carvalho, 2020; Qu et al., 2020; Sheehy, 2020). These articles were published from April 27, 2020, to July 16, 2020, and represented six institutions, including hospitals, scientific societies, and universities from five countries—United States (*n* = 1), Netherlands (*n* = 1), Canada (*n* = 2), Brazil (*n* = 1), China (*n* = 1) (see Supplementary Table 1). Of the six articles, two were published by researchers, and three were published by rehabilitation and medical professionals. The remaining article did not report the group involved in developing the recommendations.

### Summary of Key Recommendations

• Avoid transferring patients with COVID-19 into the mainstream skilled nursing facility population, as patients may still be able to transmit the disease.
• Provide discharged patients who are released from isolation to the community setting with various comprehensive rehabilitation treatment options as appropriate based on the type of dysfunction experienced by the patient.
• With regard to occupational therapists or health professionals with similar training in discharge planning:
  ◦ Prepare and plan for discharge, including home safety and caregiver supports.
  ◦ Consider social determinants of health when discharge planning (e.g., income).
• For patients discharged to home or to other facilities in the community, provide guidance on ways to manage and closely monitor physical activity.
• Develop a template for patients discharged from acute care that addresses immediate needs and rehabilitation considerations using available tools such as the Patient-Oriented Discharge Summary or Rehabilitation Prescription.

### Outpatient Rehabilitation Setting

Seven articles that addressed the outpatient rehabilitation setting (Azhari and Parsa, 2020; Boldrini et al., 2020; Ismail, 2020; Koumpouras and Helfgott, 2020; Phillips et al., 2020; Piepoli, 2020; Polastri et al., 2020) were identified. These articles, all expert opinion articles, were published from April 1, 2020, to June 5, 2020, representing five countries—Iran (*n* = 1), Italy (*n* = 3), Egypt (*n* = 1), Canada (*n* = 1), United States (*n* = 1) and seven institutions including hospitals, scientific societies, and universities (see Supplementary Table 1). Of the seven articles, three were developed by researchers, and the remaining four articles were developed by rehabilitation and medical professionals.

### Summary of Key Recommendations

• Provide instructions about social distancing and hand hygiene for both patients and staff at entrance.
• Disinfect all devices and equipment after each session.
• If there is insufficient PPE, consider cancelation of a patient appointment in the case of suspected or confirmed COVID-19.
• Introduce home and community-based physical therapy care via mobile applications to patients who would be most impacted by canceled rehabilitation or exercise sessions at outpatient clinics.

### Rehabilitation Equipment/Workplace

Four expert opinion articles addressing rehabilitation equipment and workplace (Grabowski and Joynt Maddox, 2020; Mammi et al., 2020; Sheehy, 2020; Thomas et al., 2020) were identified. Article publication dates ranged from March 30, 2020, to May 26, 2020. Five countries—[Italy (*n* = 1), Australia (*n* = 1), Belgium (*n* = 1), Canada (*n* = 2), and United States (*n* = 1)] and four institutions including hospitals, scientific societies, and universities were represented (see Supplementary Table 1). Of the four articles, two were developed by rehabilitation and medical professionals, and one was developed by researchers. The remaining article did not report the group involved in developing the recommendation.

### Summary of Key Recommendations

• Create separate working spaces e.g., separate therapy spaces, offices, gym(s), a front office, and a visitor waiting room.
• Decontaminate shared equipment between patients. Use single use equipment, when possible, e.g., Thera Bands rather than hand weights. Pay particular attention to decontaminating electrode sponges, hydrocollator heat packs, gels, topical lotions, and items for training manual dexterity.
• Identify additional physical resources required for physiotherapy interventions. Disinfect equipment to minimize the risk of cross-infection e.g., respiratory equipment; mobilization, exercise and rehabilitation equipment; and equipment storage containers.
• Identify and develop a facility inventory of respiratory, mobilization, exercise and rehabilitation equipment and determine the process of equipment allocation as pandemic levels increase (i.e., to prevent movement of equipment between infectious and non-infectious areas).

### Human Resources

Four expert opinion articles that addressed human resources in rehabilitation (Bartolo et al., 2020; Grabowski and Joynt Maddox, 2020; Sheehy, 2020; Thomas et al., 2020) were identified. These articles were published from April 30, 2020, to May 26, 2020, representing five countries [Canada (*n* = 2), Italy (*n* = 1), Australia (*n* = 1), Belgium (*n* = 1), and the United States (*n* = 1)] and four institutions including hospitals, scientific societies and universities (see Supplementary Table 1). Of the four articles, one was developed by researchers, and two were developed by rehabilitation and medical professionals. The remaining article did not report the group involved in developing the recommendations.

### Summary of Key Recommendations

• Recruit additional staff to perform tasks with attainable skills and that can be acquired relatively quickly.
• Plan for an increase in the required physiotherapy workforce. For example:
  ◦ Allow additional shifts for part-time staff.
  ◦ Offer staff the ability to electively cancel leave.
  ◦ Recruit a pool of staff available to work on an *ad hoc* basis.
  ◦ Recruit academic and research staff, and staff who have recently retired or are currently working in non-clinical roles.
  ◦ Allow for work in different shift patterns (e.g., 12-h shifts, extended evening shifts, etc.).
• Require physiotherapists to have specialized knowledge, skills and decision-making ability to work within the intensive care unit (ICU) setting. Have hospitals identify physiotherapists with previous ICU experience and ask them to return to the ICU setting.
• Have hospitals identify physiotherapists without recent cardiorespiratory physiotherapy experience and have them support additional hospital services. For example, physiotherapists without acute care or ICU training could be trained for non-clinical duties e.g., facilitate rehabilitation and discharge pathways.
• Identify existing learning resources for staff who could be deployed to the ICU setting. For example:
  ◦ eLearning packages (e.g., Clinical Skills Development Service for Physiotherapy and Critical Care Management).
  ◦ Local physiotherapy staff assistance with ICU orientation.
  ◦ PPE training.
• Ensure staff at high risk do not enter the COVID-19 isolation area. When planning for staffing and developing rosters, staff at higher risk of developing serious illness from COVID-19 should not be scheduled to work with or be exposed to infected patients; this includes staff who
  ◦ Are pregnant
  ◦ Have significant chronic respiratory illnesses
  ◦ Are immunosuppressed
  ◦ Are > 60 years of age
  ◦ Have severe chronic health conditions such as heart disease, lung disease, and diabetes.
  ◦ Have immune deficiencies, such as neutropenia, disseminated malignancy, and conditions or treatments that produce immunodeficiency.
• Include considerations for workforce planning for pandemic-specific requirements such as donning and doffing PPE and for non-clinical duties such as enforcing infection control procedures.
• Recognize that staff will likely have an increased workload and a risk of heightened anxiety at work and home. Support staff during and beyond the active treatment phases e.g., via access to employee assistance programs, counseling, and facilitated debriefing sessions.

### Telerehabilitation

Eighteen articles that addressed telerehabilitation (Besnier et al., 2020; Chang and Boudier-Revéret, 2020; D Leochico, 2020; Grabowski and Joynt Maddox, 2020; Hosey and Needham, 2020; Ismail, 2020; Juárez-Belaúnde et al., 2020; Kemps et al., 2020; Maggio et al., 2020; Pedersini et al., 2020; Piepoli, 2020; Rehabilitative Care Alliance, 2020; Tenforde et al., 2020; Verduzco-Gutierrez et al., 2020; Viswanath and Monga, 2020; Yeo et al., 2020; Zhao H. M. et al., 2020; Handu et al., 2021) were identified. Of the 18 articles, 17 were developed based on expert opinion and/or clinical experience, and one was developed using a combination of expert and/or clinical experience and evidence-based methods, including systematic review, survey, and observational studies. The articles were published from April 1 to July 16, 2020, representing 11 countries [Netherlands (*n* = 1), United States (*n* = 5), China (*n* = 1), Italy (*n* = 3), Philippines (*n* = 1), United Kingdom (*n* = 1), Canada (*n* = 2), Singapore (*n* = 1), South Korea (*n* = 1), Egypt (*n* = 1)]—and eighteen institutions including hospitals, scientific societies, and universities (Supplementary Table 1). Of the 18 articles, ten were developed by researchers and seven were developed by rehabilitation and medical professionals. The remaining article did not report the group involved in developing the recommendations.

### Summary of Key Recommendations

• Replace face-to-face sessions with remote assessment and monitoring/guidance, use telephone, text messaging, emails, video consultations, web-based platforms and applications, based on availability of local equipment and expertise.
• Use telemedicine to provide interventions typically facilitated by rehabilitation professionals e.g., physiotherapy, speech therapy, occupational therapy, patient telemonitoring, and teleconsultation, and assisting home-bound patients.
• Utilize telemedicine to provide emotional support to patients, ensure appropriate home adaptation, and prepare family members prior to discharge.
• Use telemedicine platforms supported by smartphones to increase access.
• Advise patients to complete the encounter in a location that provides privacy.
• Account for factors that impact patient comfort when performing telehealth visits. These considerations include assessment of prolonged sitting and assessing the safety of the surrounding environment to perform balance testing.
• Give healthcare providers access to relevant patient medical records before patient visits, including prior patient visit records and diagnostic testing and imaging.
• Document the visit when the clinician connects with the patient. Ensure patient identification is checked prior to the start of the visit for a new patient or for a patient without scanned identification in the medical record or patient file. In addition, obtain verbal consent from patients for telemedicine and provide a brief orientation to telemedicine at the start of the patient encounter.
• Record the patient’s location at the time of visit and gather emergency contact information. Document the chief complaint and reason for the visit and demographics (e.g., age, sex, gender, race/ethnicity).
• Encourage patients to have their medications on hand for documentation of medication reconciliation.
• Provide each patient with instructions prior to the visit on how to access the software platform. Programs can perform a “test call” with support staff to ensure the device runs the software correctly and has sufficient digital connection, ideally in the planned location for a telemedicine visit.
• Provide patient access for the visit through a secure URL link, or existing smartphone apps with a “virtual waiting room.”
• Follow a sequence mirroring an in-person visit, including identifying the chief complaint and purpose for the visit, along with relevant history.
• Use instant messaging software or apps for coordination between providers and office staff during and after the visit.
• Document the telemedicine visit similar to how an in-person visit is documented.
• Advise virtual visits during the COVID-19 pandemic to facilitate appropriate physician compensation for the visit.
• Record history of present illness, relevant past medical, surgical, family and social history; review of systems; functional status, and drug allergies.
• For physical examination, optimize observations through a video platform. Document patient instructions in narrative and descriptive format.
• Considerations when contemplating virtual care delivery of rehabilitation interventions:
  ◦ Older adult’s access to technology, internet and other limitations e.g., communication abilities.
  ◦ Potential safety issues. Engage informal caregivers to provide assistance for safety and/or for technical support.
  ◦ Type of older adult’s disability e.g., hearing or vision and its impact on their ability to participate.
  ◦ Older adult’s cognitive ability, and subsequent implications for safety, ability to complete a self-directed program and receipt of any information or instructions.
  ◦ Awareness of confidentiality issues if the older adult is living in a multi-person household. Offer to provide additional sessions via phone call or private in-person visits for sensitive issues.
  ◦ Accept digital consents or signatures for paperwork where originals are required to avoid delays because of mailing time.
  ◦ Accommodate the needs of older adults and their caregivers by being flexible.
  ◦ Allow for extra time to build rapport and trust.
  ◦ Anticipate potential technical issues.
  ◦ Provide virtual care options for psychosocial support during the in-hospital stay as a mode to enable social engagement and caregiver involvement.

### Rehabilitation Patient Precautions

Seven articles that addressed rehabilitation patients’ precautions (Bartolo et al., 2020; Mammi et al., 2020; Reuter-Oppermann et al., 2020; Romano-Bertrand et al., 2020; Sheehy, 2020; Thomas et al., 2020) were identified. These articles, all expert opinion articles, were published from March 30, 2020, to August 1, 2020, representing eight countries [Italy (*n* = 3), Germany (*n* = 1), Austria (*n* = 1), Switzerland (*n* = 1), France (*n* = 1), Australia (*n* = 1), Belgium (*n* = 1), Canada (*n* = 2)] and seven institutions including hospitals, scientific societies and universities (Supplementary Table 1). Of the seven articles, two were developed by researchers, four were developed by rehabilitation and medical professionals. The remaining publication did not report the group involved in developing the recommendations.

### Summary of Key Recommendations

• For patients using rehabilitation facility tools/gym/pool, ensure, and encourage:
  ◦ Hygiene and behavioral rules before entrance into pools.
  ◦ Use of individual dressing rooms. Use of individual dedicated compartments to hang clothes.
  ◦ Use of soap and water for showers and use booth bath when possible.
  ◦ Swim cap and swimming goggles in pools.
  ◦ Avoidance of bathers suspected of/infected with COVID-19 or with respiratory and/or digestive symptoms.
  ◦ Use of hand sanitizers at the entrance to the facility.
  ◦ Use of surgical masks until reaching the dressing room and when dressed post bathing.
  ◦ Posting of signs for a 2-m circumference physical distancing rule.
  ◦ If patients need to sneeze and/or cough, they should do so directly into their hands, then immediately wash hands with soap and water.
  ◦ Avoid touching face, nose, mouth, and eyes.

### Rehabilitation Professionals Precautions

Thirteen articles that addressed precautions for health professionals (Bartolo et al., 2020; Grabowski and Joynt Maddox, 2020; Japan ECMOnet for Covid-19, 2020; Kleinpell et al., 2020; Pandian and Sebastian, 2020; Pedersini et al., 2020; Qu et al., 2020; Reuter-Oppermann et al., 2020; Romano-Bertrand et al., 2020; Sheehy, 2020; Thomas et al., 2020; Zeng et al., 2020; Handu et al., 2021) were identified. These articles were published from March 30 to August 1, 2020 representing 11 countries [Australia (*n* = 1), Belgium (*n* = 1), Canada (*n* = 2), Germany (*n* = 1), Austria (*n* = 1), Switzerland (*n* = 1), Italy (*n* = 2), China (*n* = 2), France (*n* = 1), India (*n* = 1), and the United States (*n* = 4)] and 13 institutions including hospitals, scientific societies and universities (Supplementary Table 1). Twelve articles were developed based on expert opinion and/or clinical experience, and one was developed using evidence-based methods, including systematic review, survey and observational studies. Of the 13 publications, five were developed by rehabilitation and medical professionals, six were developed by researchers. The remaining two articles did not report the group involved in developing the recommendations.

### Summary of Key Recommendations

• Plan therapeutic activities to minimize the number of personnel involved, when possible, e.g., one therapist with a gait aid rather than a therapist and an assistant.
• Minimize the number of personnel entering a patient’s room. Have a single staff member perform most (if not all) of the care and duties for a particular patient e.g., delivery of food trays, making the bed, giving medications, and helping with morning care.
• Educate and empower all healthcare professionals involved in rehabilitation teams.
• Train all staff in correct donning and doffing of PPE, including N95 “fit-checking.” Maintain a registry of staff who have completed PPE education and fit checking.
• For healthcare workers:
  ◦ Ensure strict adherence to mitigating measures in order to prevent cross-transmission outside of pools/gyms.
  ◦ Wear a surgical mask or goggles or face shield when in close contact with patients.
  ◦ Maintain physical distancing of at least a 2-m circumference.
  ◦ Practice regular hand hygiene and avoid touching face and eyes.
• Include additional PPE precautions for staff caring for COVID-19 infected patients and/or those with significant respiratory illness, e.g., in situations when aerosol-generating procedures and/or prolonged or very close contact with the patient are likely. For all confirmed or suspected cases, implement droplet precautions at a minimum. Have staff adhere to following:
  ◦ Surgical mask, FFP2 or FFP3 mask.
  ◦ Fluid-resistant long-sleeved gown.
  ◦ Goggles or face shield.
  ◦ Gloves.
• Additional considerations for Staff:
  ◦ Hair cover for aerosol-generating procedures.
  ◦ Shoes impermeable to liquids that can be wiped down. The use of recurrent shoe covers is not recommended as repeated removal is likely to increase the risk of staff contamination.
• In dedicated units caring for a patient with confirmed or suspected COVID-19, implement patient and staff supervision of all donning and doffing by an additional appropriately trained staff member.
• Preferably only use single-use equipment and avoid sharing equipment.
• Have staff wear an additional plastic apron if a large volume of fluid exposure is expected.
• Have staff clean and disinfect any PPE items that are to be reused, e.g., goggles.
• Have staff wear scrubs and a T-shirt at work; have them shower and change into street clothes before going home.
• Have staff adhere to the following guidelines:
  ◦ Change clothes before and after work
  ◦ Shower before rejoining family.
  ◦ Limit or avoid physical contact until after showering
  ◦ Use alcohol-based hand sanitizer before entering the home.
  ◦ Shower and wash clothes away from family.
  ◦ Isolate from family members and wear a mask while at home.

## Discussion

A comprehensive summary of healthcare system related recommendations for rehabilitation services and settings (inpatient, discharge process, outpatient), logistical considerations (equipment, human resources and telerehabilitation), and precautions for both patients and rehabilitation professionals has been developed through this scoping review. The majority of the recommendations were based on expert opinions and/or consensus. The overall quality of the recommendations was determined to be fair, and most of the individual recommendations were graded as strong.

We anticipate the COVID-19 pandemic and subsequent generation of evidence will continue to evolve. Planning is underway to update the recommendations presented in this review in the near future, incorporating the most recent evidence. As COVID-19 vaccinations become more available and vaccine uptake increases across the globe, the impact of COVID-19 may lessen, and as a result the need for, and type of recommendations may change.

The impact of the COVID-19 pandemic extends beyond the number of individuals who have contracted the virus. Secondary negative effects of social isolation resulting from the required implementation of health policies for mitigating COVID-19 spread and the challenges in accessing health services are evident (Lebrasseur et al., 2021). Implementation of evidence-based and clinically relevant strategies to ensure the provision of health services for those impacted by COVID-19 is essential.

The strengths of our scoping review include pre-specified eligibility inclusion criteria, a comprehensive and up-to-date search strategy, and the inclusion of relevant articles from low-income, middle income, and high-income countries. Our review utilized duplicate assessments for eligibility determination, data extraction, evidence synthesis, and application of the GRADE method (Andrews et al., 2013; Neumann et al., 2014) in rating the strength and quality of the recommendations. Additionally, both published literature and the experiences of a multidisciplinary author team were used to develop the summary.

While this review synthesized and summarized the best available recommendations published to date, there are several limitations. We acknowledge that included recommendations were largely from expert opinion and clinical experience-based articles rather than higher-level primary studies, e.g., randomized controlled trials (RCTs). Although systematic reviews, meta-analyses, RCTs, and observational studies provide a higher level of evidence, primary studies or systematic reviews related to COVID-19 rehabilitation recommendations were not found. This is not entirely surprising considering the rapid onset and the “newness” of having to manage healthcare within the context of the COVID-19 pandemic.

In conclusion, the comprehensive summary of health-system-related rehabilitation recommendations for the global COVID-19 pandemic focuses on reducing the spread of SARS-CoV-2 infection and ensuring adequate and safe rehabilitation services, whether face-to-face or through teleservices, as appropriate. As the COVID-19 pandemic is rapidly changing, further updates are warranted in order to incorporate emerging evidence into rehabilitation guidelines.

## Author Contributions

All authors listed have made a substantial, direct, and intellectual contribution to the work, and approved it for publication.

## Conflict of Interest

YC was employed by company OrthoEvidence Inc. The remaining authors declare that the research was conducted in the absence of any commercial or financial relationships that could be construed as a potential conflict of interest.

## Publisher’s Note

All claims expressed in this article are solely those of the authors and do not necessarily represent those of their affiliated organizations, or those of the publisher, the editors and the reviewers. Any product that may be evaluated in this article, or claim that may be made by its manufacturer, is not guaranteed or endorsed by the publisher.
