# Supplementary material for: Rehabilitation Care at the Time of Coronavirus Disease-19 (COVID-19) Pandemic: A Scoping Review of Health System Recommendations
Source: Front Aging Neurosci. 2022 Jan 4;13:781271. doi: 10.3389/fnagi.2021.781271 (PMC8764235; doi:10.3389/fnagi.2021.781271)
Supplement: Supplementary file 1 [file Table_1.DOCX]

**SEARCH STRATEGY**

**Date: August 1, 2020**

1. **MEDLINE = 509**

1. exp Rehabilitation/ or exp Allied Health Personnel/ or exp Allied Health Occupations/ or (rehab* or physio or physiotherap* or physical therap* or allied health or PT).mp.

2. (Rehabilitation or Allied Health Personnel or Allied Health Occupation* or rehab* or physio or physiotherap* or physical therap* or allied health or PT).mp. [mp=title, abstract, original title, name of substance word, subject heading word, floating sub-heading word, keyword heading word, organism supplementary concept word, protocol supplementary concept word, rare disease supplementary concept word, unique identifier, synonyms]

3. exp Coronavirus/ or exp Coronavirus Infections/ or coronaviru*.mp. or "corona virus*".mp. or ncov*.mp. or n-cov*.mp. or "novel cov".mp. or COVID-19.mp. or COVID19.mp. or COVID-2019.mp. or COVID2019.mp. or SARS-CoV-2.mp. or SARSCoV-2.mp. or SARSCoV2.mp. or SARSCoV19.mp. or SARS-Cov-19.mp. or SARSCov-19.mp. or SARSCoV2019.mp. or SARS-Cov-2019.mp. or SARSCov-2019.mp. or "severe acute respiratory syndrome coronaviru*".mp. or "severe acute respiratory syndrome cov 2".mp. or "2019 ncov".mp. or 2019ncov.mp.

4. 1 or 2

5. 3 and 4

1. **PUBMED = 511**

**Pneumonia, Viral/or Coronavirus Infection or Betacoronavirus**

- "coronavirus"[MeSH Terms] OR "coronavirus infections"[MeSH Terms] OR "coronaviru*"[Title/Abstract] OR "corona virus"[Title/Abstract] OR "ncov*"[Title/Abstract] OR "n cov*"[Title/Abstract] OR "novel cov"[Title/Abstract] OR "COVID-19"[Title/Abstract] OR "COVID19"[Title/Abstract] OR "COVID-2019"[Title/Abstract] OR "COVID2019"[Title/Abstract] OR "SARS-CoV-2"[Title/Abstract] OR "SARSCoV-2"[Title/Abstract] OR "sarscov2"[Title/Abstract] OR "SARSCoV19"[Title/Abstract] OR "sars cov 19"[Title/Abstract] OR "severe acute respiratory syndrome cov2"[Title/Abstract] OR "2019 ncov"[Title/Abstract] OR "2019ncov"[Title/Abstract] OR "severe acute respiratory disease"[Title/Abstract]

**Rehabilitation**

- "rehabilitation"[MeSH Terms] OR "allied health personnel"[MeSH Terms] OR "allied health occupations"[MeSH Terms] OR "rehab*"[Title/Abstract] OR "physio"[Title/Abstract] OR "physiotherap*"[Title/Abstract] OR "physical therap*"[Title/Abstract] OR "allied health"[Title/Abstract] OR "PT"[Title/Abstract]

1. **CINAHL = 177**

"coronavirus" OR "coronavirus infections" OR "coronaviru*" OR "corona virus" OR "ncov*"[Title/Abstract] OR "n cov*"[Title/Abstract] OR "novel cov"[Title/Abstract] OR "COVID-19"[Title/Abstract] OR "COVID19"[Title/Abstract] OR "COVID-2019" OR "COVID2019" OR "SARS-CoV-2" OR "SARSCoV-2" OR "sarscov2" OR "SARSCoV19" OR "sars cov 19" OR "severe acute respiratory syndrome cov2" OR "2019 ncov" OR "2019ncov" OR "severe acute respiratory disease"

rehabilitation or "allied health personnel" or "allied health occupations" or "rehab*" or "physio" or "physiotherap*" or "physical therap*" or "allied health" or "PT" or "OT"

1. **Embase = 2022**

1. exp Rehabilitation/ or exp Allied Health Personnel/ or exp Allied Health Occupations/ or (rehab* or physio or physiotherap* or physical therap* or allied health or PT).mp.

2. (Rehabilitation or Allied Health Personnel or Allied Health Occupation* or rehab* or physio or physiotherap* or physical therap* or allied health or PT).mp. [mp=title, abstract, original title, name of substance word, subject heading word, floating sub-heading word, keyword heading word, organism supplementary concept word, protocol supplementary concept word, rare disease supplementary concept word, unique identifier, synonyms]

3. exp Coronavirus/ or exp Coronavirus Infections/ or coronaviru*.mp. or "corona virus*".mp. or ncov*.mp. or n-cov*.mp. or "novel cov".mp. or COVID-19.mp. or COVID19.mp. or COVID-2019.mp. or COVID2019.mp. or SARS-CoV-2.mp. or SARSCoV-2.mp. or SARSCoV2.mp. or SARSCoV19.mp. or SARS-Cov-19.mp. or SARSCov-19.mp. or SARSCoV2019.mp. or SARS-Cov-2019.mp. or SARSCov-2019.mp. or "severe acute respiratory syndrome coronaviru*".mp. or "severe acute respiratory syndrome cov 2".mp. or "2019 ncov".mp. or 2019ncov.mp.

4. 1 or 2

5. 3 and 4
