# Supplementary material for: Rehabilitation Care at the Time of Coronavirus Disease-19 (COVID-19) Pandemic: A Scoping Review of Health System Recommendations
Source: Front Aging Neurosci. 2022 Jan 4;13:781271. doi: 10.3389/fnagi.2021.781271 (PMC8764235; doi:10.3389/fnagi.2021.781271)
Supplement: Supplementary file 2 [file Table_2.DOCX]

| **System-related recommendations** | | **Direction and strength of recommendations** | |
| --- | --- | --- | --- |
| **No.** | **Rehabilitation facility/divisions/inpatients** |  |  |
| 1 | Control the entrance of patients to Rehabilitation institutions.  Establish standardized pre-examination triage points in outpatient and emergency departments. | Strong For |  |
| 2 | Ensure institutions formulate screening procedures for patient admission during the pandemic in accordance with the risk level of the country and region.  Expedite screening and diagnosis of suspected patients, implement isolation measures quickly, and shorten the time between diagnosis and hospitalization. | Strong For |  |
| 3 | Ensure notices with behavioral rules at the entrances to and within all the departments are posted.  Ensure hand hygiene recommendations near hand-sanitizing gel dispensers are posted. | Strong For |  |
| 4 | Limit admissions to rehabilitation wards to essential workers supervised by personnel equipped with personal protective equipment (PPE). Close all other accesses. | Strong For |  |
| 5 | Perform imaging and laboratory testing of suspected COVID-19 cases as early as possible; this can be achieved by focusing on relevant clinical manifestations, epidemiological history, and temperature monitoring. | Strong For |  |
| 6 | Treat patients in a single room before SARS-CoV-2 infection is ruled out, then transfer patients to a conventional ward for further hospitalization. | Strong For |  |
| 7 | Maintain a space of more than one meter between beds in the general ward to reduce the potential risk of cross-infection in the hospital. | Strong For |  |
| 8 | Proactively monitor nosocomial infections in hospitalized patients by establishing health monitoring and a mandatory reporting system for all members of the hospital personnel (including all medical, nursing, management, logistics, cleaning, security, delivery, and other staff). | Strong For |  |
| 9 | Actively monitor fever, respiratory symptoms, and chest imaging to promptly detect cases of sporadic infections, clustered infections, and suspected infections, allowing for appropriate and timely investigations and preventive measures. | Strong For |  |
| 10 | Suspend caregiver visits to hospitalized patients except in certain circumstances.  Authorize exceptional cases by clinicians according to the rules of the health management; manage access in derogation by the staff in order to avoid any contact and for a limited time.  Screen escorts and visitors and manage them as necessary. Monitor body temperature of all health personnel and visitors with permission; prohibit access and give an indication of home isolation if body temperature is higher than 37.5°C.  Offer virtual visits. | Weak For |  |
| 11 | Ensure the doors of Rehabilitation units are closed in order to control and record (indicating the time) the flow of all the entrances. | Strong For |  |
| 12 | Suspend o all meeting activities and replaced by telephone, email contact, or any virtual meetings tools. | Strong For |  |
| 13 | Conduct clinical interviews with family members by phone, email, or any virtual meetings tools. | Strong For |  |
| 14 | Suspend all rehabilitation activities that require internal flow (movement between floors or to reach gyms) for patients with COVID-19. | Strong For |  |
| 15 | For patients with COVID-19, prohibit group therapy and therapy in rehabilitation gyms; provide therapy one-on-one in patients' rooms. | Strong For |  |
| 16 | Transform all nonessential treatments into a telerehabilitation/virtual reality modality and manage clinical cases through telephone or webcam counselling; provide supervision to exercise sessions that can be temporarily self-managed by the patient or caregiver. | Strong For |  |
| 17 | Reduce the rehabilitation team activities (keep only strictly necessary as carried out by the clinician in consult with the coordinator and with the other staff). | Weak For |  |
| 18 | Re-evaluate the possibility of the epidemiological evolution of the pandemic and of the reintroduction of certain contact situations (with appropriate PPE and devices) in circumstances that may require urgent hands-on treatment; this may be necessary to prevent the health of the patient from degenerating due to hypo-mobility, respiratory dysfunction, or from contextual factors. | Weak For |  |
| 19 | Carry out rehabilitation activities in the patients' room where possible; in gym activities, strictly keep the distance of at least 2 meters between the patients. | Strong For |  |
| 20 | Ensure specialized neurorehabilitation units and their facilities and resources are adapted to enable better management of both patients with sequelae after SARS-CoV-2 infection and their usual patients. | Weak For |  |
| 21 | Develop specialized post-acute care environments to treat patients who are recovering from COVID-19 and for those who may be potentially contagious who cannot receive care at existing facilities. | Strong For |  |
| 22 | Consider receiving patients from acute care earlier than is generally done. | Strong For |  |
| 23 | Increase the admission capacity of rehabilitation facilities to support early discharge from the acute care units and facilitate the early subsequent safe discharge to outpatient and home and community rehabilitation services whenever possible. | Strong For |  |
| 24 | Enforce daily cleaning and disinfection of environmental surfaces, particularly in terms of disinfection frequency of high-frequency contact surfaces (i.e., door handles, computer keyboards, and instrument panels).  Prepare plans for extraordinary sanitization and cleaning in cases of access or identification of a "suspected case." | Strong For |  |
| 25 | Emphasize the role of remote assistance/home monitoring and/or telerehabilitation in advance for patients who are unable to access rehabilitation hospitals or need to be discharged for consulting activities in hospitals or outpatients consults. | Strong For |  |
| 26 | Reinforce indoor ventilation and mechanical ventilation if necessary. Implement "No-touch" disinfection methods, such as ultraviolet light devices or hydrogen peroxide systems, to reduce the cleaning workload of hospital staff. | Weak For |  |
| 27 | Suspend centralized air conditioning and ventilation systems during an epidemic for increased prevention and control.  Completely close the air return valve when it is necessary to open the centralized air conditioning ventilation system. Completely open the fresh air valve to increase the fresh air volume in the system.  For centralized air conditioning ventilation systems, turn on the corresponding exhaust system and equip the air return port with a nanometer or high-intensity ultraviolet lamp and other disinfection devices. Regularly clean and disinfect all components of the centralized air conditioning ventilation system and replace components once per month and again after the pandemic has ended. | Strong For |  |
| 28 | Implementation of sewage system disinfection measures for the hospital logistics department in accordance with relevant national regulations is a mandatory requirement. Manage medical waste generated by patients diagnosed with or suspected to have COVID-19, including the domestic waste of these patients as infectious medical waste and strictly dispose it in accordance with relevant regulations. | Strong For |  |
| 29 | Ensure staff of external companies comply rigorously and systematically with standard precautions in addition to those provided by air, by droplets, and by contact, as indicated in the behavioral rules. | Strong For |  |
|  | **Outpatients** |  |  |
| 30 | For chronic disabling conditions, with or without exacerbations, and for persons who did not suffer from a recent acute event, consider preliminarily alternative options of care (e.g., remote consultation, telerehabilitation) to postpone the treatments while maintaining the therapeutic relationship. Exceptions to such behavior may be chronic conditions at risk of rapid deterioration of functional level when left untreated (e.g., neurodegenerative diseases, severe conditions in childhood). | Weak For |  |
| 31 | Ask patients to call ahead of their scheduled appointment. On the phone, ask about fever, respiratory symptoms, family contact with a case of COVID-19, or recent travel to evaluate the need to postpone any upcoming appointments. | Strong For |  |
| 32 | Re-evaluate the reasons for an appointment to find out suitable patients for telerehabilitation or home-based care. | Strong For |  |
| 33 | At the entrance, provide instructions about social distancing and hand hygiene for both patients and staff. | Strong For |  |
| 34 | Provide alcoholic sanitizer or hand rub solutions. Ensure tissues are available at the check-in front desk and in the waiting area. | Strong For |  |
| 35 | Use plastic/glass barriers to minimize the contact between staff and patients. | Strong For |  |
| 36 | Disinfect all devices and equipment after each session. | Strong For |  |
| 37 | Consider cancellation of appointment if PPE is not enough to admit a suspected or confirmed case of COVID-19. | Strong For |  |
| 38 | Considering priority and elective cases, reduce the number of outpatient appointments by using the capacity of telerehabilitation, as this would help to establish a one-by-one service. | Strong For |  |
| 39 | Consider offering consultations via video without the need to see the patient in person; gather most of the patient history via telephone or video with the primary team members. | Strong For |  |
| 40 | In light of less effective, highly expensive home mobile rehabilitation applications and the concurrent closure of outpatient clinics of PT in hospitals, health clubs and gyms, introduce and provide home and community-based care (HCBC) physical therapy (PT) to patients for those who may be harmed by cancelled exercise sessions. | Strong For |  |
| 41 | Consider physical, cognitive, and psychosocial outcomes for any rehabilitative activities provided in an outpatient setting. | Strong For |  |
|  | **Discharge** |  |  |
| 42 | Transferring patients with COVID-19 into the mainstream skilled nursing facility population may not be safe for hospitals. In certain cases, some patients may be able to transmit the disease still. | Weak For |  |
| 43 | Support acute cardiac wards to provide a summary of important information/recommendations on secondary prevention (including information on physical activity and mental health) before hospital discharge. | Strong For |  |
| 44 | Ensure patients who are not immunosuppressed continue to self-isolate (14 days for patients returning to long-term care facilities or other congregate group homes) after illness onset (whichever is longer). Ensure staff continue to use PPE) until their predominant symptoms and fever are completely resolved or until ten days. | Strong For |  |
| 45 | Ensure PT help with the post-hospital discharge rehabilitation program. | Strong For |  |
| 46 | Provide discharged patients to the community who are released from isolation with various forms of comprehensive rehabilitation treatment as appropriate to the types of dysfunctions experienced by the patients. | Strong For |  |
| 47 | Consideration for the role of occupational therapy or health professionals with similar training in discharge: | Strong For |  |
|  | - Provide preparation and planning for discharge, including home safety and caregiver supports. | Strong For |  |
|  | - Incorporate social determinants of health in discharge planning (e.g., income). | Strong For |  |
| 48 | Provide patients discharged for home or other facilities in the community with indications on how to cope with the physical activity (PA); ensure PA is closely monitored regarding function, capacity, and participation when the patient is cured and is no longer at risk of the contagion or the virus. | Strong For |  |
| 49 | Consider discharging patients to home sooner than usual to free space (as soon as the family is able to take care of the patient). | Weak For |  |
| 50 | Ensure critically ill COVID-19 patients are provided with extensive and prolonged rehabilitation after post-acute care discharge in the appropriate setting to address cognitive, psychological, and physical impairments resulting from the infection, mechanical ventilation and ICU stay.  Consider developing a template for patients discharged from acute care to addresses immediate needs and rehabilitation considerations using available tools such as the Patient-Oriented Discharge Summary or Rehabilitation Prescription. | Strong For |  |
|  | **Rehabilitation Equipment/working space** |  |  |
| 51 | Create working spaces for rehabilitation needs such as occupational therapist and speech therapist office, gyms, a front office, and a visitor waiting room. | Strong For |  |
| 52 | Ensure sufficient standard PPE for procedures and needs are identified ahead of time, as hospital resources diminish quickly.  Provide staff with appropriate safety equipment and training to deliver rehabilitative care. | Strong For |  |
| 53 | Decontaminate shared equipment between patients; use single-use equipment wherever possible (e.g., Thera Bands rather than hand weights). Pay particular attention to electrode sponges, hydrocollator heat packs, gels, topical lotions, and items for training manual dexterity. | Strong For |  |
| 54 | Identify additional physical resources that may be required for physiotherapy interventions and how the risk of cross-infection can be minimised (e.g., respiratory equipment, mobilisation, exercise and rehabilitation equipment, and equipment storage). | Strong For |  |
| 55 | Identify and develop a facility inventory of respiratory, mobilization, exercise, and rehabilitation equipment. Additionally, determine the process of equipment allocation as pandemic levels increase (i.e., to prevent movement of equipment between infectious and non-infectious areas). | Strong For |  |
|  | **Human Resources** |  |  |
| 56 | Recruit additional staff from industries who may experience major layoffs in the near term and can be acquired relatively quickly to perform lower-skilled tasks. | Weak For |  |
| 57 | Plan for an increase in the required physiotherapy workforce. For example:  -allow additional shifts for part-time staff  -offer staff the ability to cancel leave electively  -recruit a pool of casual staff  -recruit academic and research staff and staff who have recently retired or are currently working in non-clinical roles.  -staff that can work different shift patterns (e.g., 12-hour shifts, extended evening shifts.) | Weak For |  |
| 58 | Identify, prioritize, and deploy additional staff with previous cardiorespiratory and critical care experience to areas of higher activity associated with COVID-19 admissions (e.g., infectious disease ward, ICU and/or high dependency unit and other acute areas). | Weak For |  |
| 59 | It is required for PTs to have specialized knowledge, skills, and decision-making ability to work within ICU. Ensure hospitals identify physiotherapists with previous ICU experience and facilitate their return to the ICU. Additionally, have hospitals identify physiotherapists with no recent cardiorespiratory physiotherapy to facilitate their return to support additional hospital services. For example, staff without acute care or ICU training may facilitate rehabilitation, discharge pathways or hospital avoidance for patients without COVID-19. | Strong For |  |
| 60 | Support staff with advanced ICU physiotherapy skills to screen patients with COVID-19 assigned to physiotherapy caseloads and provide junior ICU staff with appropriate supervision and support, particularly with decision-making for complex patients with COVID-19. Ensure hospitals identify appropriate physiotherapy clinical leaders to implement this recommendation. | Strong For |  |
| 61 | Identify existing learning resources for staff who could be deployed to ICU. For example:  -eLearning packages (e.g., Clinical Skills Development Service for Physiotherapy and Critical Care Management).  -local physiotherapy staff ICU orientation.  -PPE training. | Strong For |  |
| 62 | Keep staff informed of plans. Communication is crucial to the successful delivery of safe and effective clinical services. | Strong For |  |
| 63 | Ensure staff at high risk do not enter the COVID-19 isolation area. When planning staffing and rosters, the following staff may be at higher risk of developing more serious illness from COVID-19; ensure high-risk staff are not exposed to patients with COVID-19. This includes staff who:  -are pregnant.  -have significant chronic respiratory illnesses.  -are immunosuppressed.  -are older (e.g., > 60 years).  -have severe chronic health conditions such as heart disease, lung disease, and diabetes.  -have immune deficiencies, such as neutropenia, disseminated malignancy and conditions or treatments that lead to immunodeficiency. | Strong For |  |
| 64 | Identify hospital-wide plans for allocation/cohorting patients with COVID-19. Utilize these plans to prepare required resource plans. | Strong For |  |
| 65 | Consider organising the workforce into teams that will manage COVID-19 versus non-infectious patients. Minimize or prevent movement of staff between teams. Liaise with local infection control services for recommendations. | Weak For |  |
| 66 | Consider dividing rehabilitation staff into two teams who work independently of each other so that if several members of one team become ill, the other team can take over. | Weak For |  |
| 67 | Where possible, encourage staff to work from home (e.g., administrative activities, social workers.). | Strong For |  |
| 68 | Include considerations for workforce planning for pandemic-specific requirements such as additional workload from donning and doffing PPE and the need to allocate staff to key non-clinical duties such as enforcing infection control procedures. | Strong For |  |
|  | Recognize staff will likely have an increased workload with a heightened risk of anxiety both at work and home. Support staff during and beyond the active treatment phases (e.g., access to employee assistance programs, counselling and facilitated debriefing sessions).  Consider and/or promote debriefing and psychological support; staff morale may be adversely affected due to the increased workload, infection, death of coworkers, anxiety over personal safety and the health of family members. |  |  |
| 69 | Ensure everyone is obligated to report any symptoms that arise and respect home isolation when any symptoms arise. | Strong For |  |
|  | **Telerehabilitation** |  |  |
| 70 | Replace face-to-face sessions with remote assessment and monitoring/guiding, according to local equipment and expertise (e.g., telephone, text messaging, emails, video consultations, web-based platforms, and applications). | Strong For |  |
| 71 | Increase access to facility and home care settings via telemedicine to help prevent the spread of the disease by eliminating in-person contact. | Strong For |  |
| 72 | Provide educational videos, self-management booklets, and remote consultations for patients in isolation wards during respiratory rehabilitation to reduce the usage of protective equipment and to avoid cross-infection. | Strong - For |  |
| 73 | Transform nonessential treatments into a telerehabilitation modality and manage clinical cases through telephone or webcam counselling to supervise exercise sessions that can be temporarily self-managed by the patient or caregiver. | Strong For |  |
| 74 | Utilize telerehabilitation to deliver interventions such as physiotherapy, speech therapy, occupational therapy, patient telemonitoring, and teleconsultation to assist home-forced patients without the physical presence of therapists or other healthcare professionals.  Additionally, provide emotional support to patients via teleconference prior to discharge to ensure appropriate home adaptation and to prepare family members for caregiving. | Strong For |  |
| 75 | Encourage performing a wide range of exercises, such as video or app-guided equipment-free aerobics or strength training, at home post-discharge | Strong For |  |
| 76 | Continue to strengthen present and future telerehabilitation endeavours via multidisciplinary collaboration with referring medical specialties, information and communication technology experts, data privacy officers, and medicolegal lawyers, among others. | Strong For |  |
| 77 | Utilize platforms supported by smartphones for practical reasons. | Strong For |  |
| 78 | Ensure the physiatrist has adequate technical support staff to arrange logistics prior to the telemedicine visit. | Weak For |  |
| 79 | Provide education and training recognizing physiatrists are likely to feel most comfortable delivering telemedicine by their fourth encounter. | Weak For |  |
| 80 | Have patients complete the encounter in a location that provides appropriate privacy. | Weak For |  |
| 81 | Ensure patient comfort is taken into consideration when performing visits that may require sitting for prolonged discussion or when assessing the safety of the surrounding environment (e.g.to perform balance testing). | Strong For |  |
| 82 | Ensure access to relevant medical records, including prior visit records, diagnostic testing, and imaging prior to patient visits. | Strong For |  |
| 83 | The visit begins when the physician connects with the patient. Check patient's identification prior to the start of a visit, particularly for a new patient.  Obtain verbal consent for telemedicine from patients without their scanned identification in the file or medical record. Provide a brief orientation to telemedicine at the start of the encounter.  Record patient location at the time of visit and emergency contact information. Document the chief complaint and reason for the visit along with age, sex, gender, and race/ethnicity.  Ensure patients have their medications handy for any medication reconciliation. | Strong For |  |
| 84 | Provide each patient with instructions prior to the visit on how to access the software platform. Some programs can perform a "test call" with support staff to ensure the device runs the software correctly and has sufficient digital connection in the location planned for a telemedicine visit. | Strong For |  |
| 85 | Ensure the patient accesses the visit through a secure URL link or online Website, with a "virtual waiting room," or is contacted by the physician directly via existing smartphone apps. | Strong For |  |
| 86 | Follow a normal sequence similar to an in‐person visit for identifying the chief complaint, the purpose for a visit, and for collecting any relevant patient history. | Strong For |  |
| 87 | Use instant messaging software or apps for care coordination between the providers and the office staff during and after the patient visit. | Strong For |  |
| 88 | Ensure the practice for documenting a telemedicine visit is in a format similar to that of an in‐person visit. | Strong For |  |
| 89 | Inform patients visit is being conducted virtually to facilitate compensation during the pandemic. | Strong For |  |
| 90 | Record history of present illness or interval history and identify other key aspects such as past medical, surgical, family history, review of systems, functional status, family history, social history, and drug allergies. | Strong For |  |
| 91 | Ensure the physical examination reflects observations are augmented through video platform and patient instructions and documented in a more narrative and descriptive format than the traditional office note. | Strong For |  |
| 92 | As with in‐person visits, identify the assessment and plan. | Strong For |  |
| 93 | Use standard language in consult with the institution at the conclusion of the note to reflect the visit was conducted via a telemedicine platform. | Strong For |  |
| 94 | Incorporate features from current practice of in‐person telemedicine visits. | Strong For |  |
| 95 | Maintain the same level of professionalism for physicians and environment in telemedicine as an in‐person clinical encounter. | Strong For |  |
| 96 | Ensure the physician is dressed professionally and appropriately groomed. | Strong For |  |
| 97 | Minimize background noise and announce presence at the time of the encounter to respect patient privacy. | Strong For |  |
| 98 | Display a video frame to allow the physician to preview what the patient is seeing. Ensure the physician is contained within the video frame, similar to when taking a photograph (e.g., not having a portion of the head cut off). | Strong For |  |
| 99 | Although it is tempting to look at the screen, remember to look into the video camera lens to allow the patient to experience direct eye contact.  Maintaining eye contact and body cues are important nonverbal forms of communication with the patient. | Strong For |  |
| 100 | Share results in a manner consistent with best clinical practice. | Strong For |  |
| 101 | For imaging review, share the actual image to enhance the patient experience. | Strong For |  |
| 102 | When screen sharing, ensure nonessential or private information is not inadvertently shared. | Strong For |  |
| 103 | Physicians can use video to demonstrate physical examination maneuvers that the patient is instructed to perform. | Strong For |  |
| 104 | Ensure professionals maintain appropriate rehabilitative and regulatory standards of care. | Strong For |  |
| 105 | In addition, display home exercises with proper mechanics using the video platform cue exercises using audio and visual feedback. | Strong For |  |
| 106 | Ensure the patient has the capacity, a straightforward treatment request, and the clinician has all the necessary patient information at hand. | Strong For |  |
| 107 | In low-risk patients, virtual home-based cardiac rehabilitation (VCR) can be an alternative to rehabilitation in a specialized center. | Strong For |  |
| 108 | Encourage the use of innovative VCR programs with remote monitoring trackers to help cardiac patients manage their heart disease, manage medications (therapeutic education), promote a healthy diet, and increase physical activity. | Strong For |  |
| 109 | Advise the use of trackers to quantify physical activity in patients, leading patients to adopt an active lifestyle while ensuring safety. | Weak For |  |
| 110 | Ensure patients can contact the health care team at any time. Ensure the interface can record and send variables (e.g., energy expenditure, body mass, glycemia, blood pressure, heart rate, electrocardiogram [ECG]) as measured via sensors to a web platform accessible to the physician, cardiologist, exercise specialists, and nurses. | Strong For |  |
| 111 | Consider prescribing exercise remotely and any time in selected patients suitable for home-based CR (HBCR)with the advent of smartphone applications and wearable activity trackers. | Strong For |  |
| 112 | With telerehabilitation, a physician can determine whether a patient needs to be tested for COVID-19 and can help avoid unnecessary hospital visits when the patient does not need a test. | Strong For |  |
| 113 | Utilize telerehabilitation for stroke patients to reduce the risk of infection, to check for changes in symptoms, and to quickly detect symptom exacerbation to ensure timely treatment.  Ensure stroke patients with COVID-19 who are asymptomatic or have mild symptoms are in self-quarantine at home. | Strong For |  |
| 114 | Utilize special coordination, such as nutrition screening using patient-room telephones to minimize staff exposure. | Weak For |  |
| 115 | Adapt telehealth as an important adaptation to deliver post-discharge assessments and interventions. | Strong For |  |
| 116 | Encourage and supervise home exercise in older adults for exercise adherence. Have PTs create at least 3- week sessions of scheduled live grouped exercise videos across the internet that can be made available via free mobile video conference applications.  These applications may be downloaded and prepared on smartphones for technologically challenged older adults with the assistance of family members and caregivers.; | Strong For |  |
| 117 | Consider the following factors for virtual care delivery of rehabilitation interventions:  -Older adult's access to technology, internet, and other practical limitations (e.g., communication abilities).  -Potential safety issues. Engage informal caregivers to assist with the safety of tasks during the intervention and/or for technical support.  -When older adults have difficulty with hearing or vision as this can impact their ability to participate.  -Older adult's cognitive ability may impact their safety, ability to complete a self-directed and to adhere to any advice that is provided.  -Confidentiality issues. Older adults with disabilities may be at home with other caregivers, which can have implications for confidentiality.  -Additional sessions may be required for sensitive issues, e.g., phone calls or in-person visits.  -Clients may need to sign consents and/or paperwork where originals are required. Additional time for mailing may be needed.  - Provide flexible hours to accommodate the needs of the older adults and their caregivers. Allow extra time  to build rapport and trust and for any technical issues, which can take time.  -Provide virtual care options for psychosocial support during an in-hospital stay as a mode to enable social engagement  and caregiver involvement.  -Ensure virtual care includes digital supports for self-care, online education, self-management tools, provider-to-provider and provider-to-  patient supports via messaging, email, text, and remote home monitoring | Strong For |  |
|  | **Patient Precautions** |  |  |
| 118 | Provide information on any changes or restrictions to the entrances and waiting areas and disinfection stations online and via telephone for patients and visitors. | Strong For |  |
| 119 | Screen all patients with COVID-19 for physiotherapy. Use telephones as screening tools whenever feasible. | Strong For |  |
| 120 | Subject visitors to careful triage by explicitly asking where they came from and detecting potential contacts even in the absence of fever. | Strong For |  |
| 121 | For patients using rehab facility tools/gym/pool  -Ensure good hygiene and good behavioural practices and rules before entrance into pools  -Encourage use of individual dressing rooms  -Provide hangers in individual dedicated compartments  To hang and store clothes.  -Use soap and water for a shower before and after bathing  -Booth bath  -Wear a swim cap  -Wear swimming goggles  -Ensure bathers suspected of/affected with COVID-19 are avoided.  - Restrict access to bathers presenting respiratory and/or digestive symptoms  -Ensure bathers follow strict measures of barriers to prevent cross-transmission outside pools.  -Ensure strict hand hygiene at the entrance to the building and while the bathroom. Used.  -Advise use of surgical masks in the hallways and for entrance to the dressing room and after post bathing and dressing.  -Ensure physical distancing of at least two metres is practices.  -Ensure any Sneezing and coughing is done directly onto hands and ensure hands are then washed with soap and water  -Avoid touching of face, nose, mouth, and eyes. | Strong For |  |
| 122 | If adequate PPE is not available, patients should stay in their rooms. | Strong For |  |
| 123 | Patients with confirmed or suspected COVID-19 will be managed with either droplet or airborne precautions and will be placed in isolation. | Strong For |  |
| 124 | Patients should be kept at least 2 meters apart and avoid talking or eating while facing each other. | Strong For |  |
| 125 | Strengthen patient and caregiver support networks through phone calls or other communication technologies. | Strong For |  |
|  | **Precautions for Health Professionals** |  |  |
| 126 | Plan therapeutic activities to minimize the number of personnel involved when possible (e.g., one therapist with a gait aid rather than a therapist and an assistant).  Minimize the number of personnel entering a patient's room. Have a single staff member perform most (if not all) of the care and duties for a particular patient (e.g., delivering food trays, making the bed, delivering medications, helping with morning care). | Strong For |  |
| 127 | Avoid routine entering of physiotherapy staff in isolation rooms where patients confirmed or suspected of COVID-19 are isolated or cohorted, except in case of screening referrals. | Strong For |  |
| 128 | Reduce meetings and physical contact with patients; ensure the greater distance between employee seats; provide hygiene training for employees and any employees with the home office. | Strong For |  |
| 129 | Empower by educating all healthcare professionals involved in rehabilitation teams by providing specific targeted training (e.g., about the correct use of PPE).  Train all staff in correct donning and doffing of PPE, including N95' fit-checking'. Maintain a registry of staff who have completed PPE education and fit checking. | Strong For |  |
| 130 | Recommend enhanced hand hygiene in accordance with World Health Organization (WHO) guidelines for all healthcare professionals before and after each with a patient and whenever hands move from one patient to another surface. | Strong For |  |
| 131 | Ensure masks with greater protection (FFP2, FFP3) are used only in suspicious or full-blown cases and not abused to reduce their availability for cases with a real need. | Strong For |  |
| 132 | For healthcare workers, ensure  -strict respect of barrier measures in order to prevent cross-transmission outside of pools/gym  - surgical mask-wearing  - physical distancing of at least 2 meters  -regular use of hand hygiene  - touching of ace and eyes are avoided  - goggles or face shields are worn when in close contact with any patient. | Strong For |  |
| 133 | Recommend PPE for staff caring for COVID-19-infected patients includes added precautions for patients with significant respiratory illness when aerosol-generating procedures are likely and/or prolonged or very close contact with the patient is likely. For all confirmed or suspected cases, implement droplet precautions at a minimum. Ensure staff wear the following items:  -surgical mask, FFP2 or FFP3 mask  -fluid-resistant long-sleeved gown  -goggles or face shield  -gloves  In addition, the following items for staff are recommended:  -hair cover for aerosol-generating procedures  -shoes impermeable to liquids that can be wiped down.  Lastly, avoid recurrent use of shoe covers as repeated removal is likely to increase the risk of staff contamination. | Strong For |  |
| 134 | Supervise all donning and doffing by an additional appropriately trained staff member when a unit is caring for a patient with confirmed or suspected COVID-19.  Additionally, avoid sharing equipment. If preferable, only use single-use equipment.  Wear an additional plastic apron if a large volume of fluid exposure is expected.  If reusable PPE items are used (e.g., goggles), clean and disinfect prior to re-use. | Strong For |  |
| 135 | Keep PPE in place, wear PPE correctly for the duration of exposure to potentially contaminated areas. Ensure PPE (particularly masks) are not adjusted during patient care. | Strong For |  |
| 136 | Test staff regularly to ensure staff are not spreading the virus. | Strong For |  |
| 137 | Ensure allied health professionals wear scrubs and a T-shirt at work and shower and change into street clothes before going home.  Ensure viral burden on clothing is reduced by following hygiene measures upon returning home and practicing distancing from family members.  Encourage changing clothes before and after work before travel to home, shower before rejoining family, limit or avoid physical contact with the family until after showering, use alcohol-based hand sanitizer before entering the house, shower, and wash clothes away from household and family use washers and dryers, isolate from family members while at home, isolate from family members by living in alternate housing, and by wearing a mask while at home. | Strong For |  |
| 138 | Minimize personal effects in the workplace. Remove all personal items before entering clinical areas and donning PPE. Personal items include earrings, watches, lanyards, mobile phones, pagers, pens. Tie back hair to keep it out of the face and eyes. | Strong For |  |
| 139 | Involve senior PTs in determining the appropriateness of physiotherapy interventions for patients with confirmed or suspected COVID-19 in consultation with senior medical staff and according to referral guidelines. | Strong For |  |
| 140 | Require continuous staff training to keep up with changing protocols/guidelines. | Strong For |  |
| 141 | To manage increasingly complex patients with COVID-19 who require high levels of ED care, consider fostering interdisciplinary collaborations with orthopedic-trained physicians to set up urgent care spaces specific to musculoskeletal injuries to reduce ED crowding, for utilizing physical therapy clinic spaces, and in helping free up hospital resources. | Strong For |  |
